# Estimated Prevalence of Asthma in US Children With Developmental Disabilities

**DOI:** 10.1001/jamanetworkopen.2020.7728

**Published:** 2020-06-16

**Authors:** Luyu Xie, Andrew Gelfand, George L. Delclos, Folefac D. Atem, Harold W. Kohl, Sarah E. Messiah

**Affiliations:** 1 School of Public Health, University of Texas Health Science Center at Houston, Dallas Campus, Dallas; 2 Center for Pediatric Population Health, Children’s Health System of Texas, Dallas; 3 Department of Pediatrics, University of Texas Southwestern Medical Center, Dallas; 4 School of Public Health, University of Texas Health Science Center at Houston, Houston campus, Houston; 5 School of Public Health, University of Texas Health Science Center at Houston, Austin campus, Austin

## Abstract

**Importance:**

The prevalence of asthma in US children with various developmental disabilities and delays is unclear, including how estimates vary by ethnic group.

**Objective:**

To report asthma prevalence estimates by various disability categories and developmental delays in a diverse sample of the US pediatric population.

**Design, Setting, and Participants:**

This population-based cross-sectional study encompassed a total of 71 811 families with children or adolescents aged 0 to 17 years (hereinafter referred to as children) who participated in the 2016 and 2017 National Survey of Children’s Health. Data were collected from June 10, 2016, to February 10, 2017, for the 2016 survey and from August 10, 2017, to February 10, 2018, for the 2017 survey. Data were analyzed from September 20, 2019, to April 5, 2020.

**Exposures:**

Developmental disability, including attention-deficit/hyperactivity disorder, autism spectrum disorder, cerebral palsy, seizure, intellectual and/or learning disability, and vision, hearing, and/or speech delay. Delay was defined as not meeting growth milestones with unknown cause.

**Main Outcomes and Measures:**

Weighted asthma prevalence estimates and 95% CIs were generated for children with and without disabilities.

**Results:**

A total of 71 811 participants (mean [SE] age, 8.6 [0.1] years; 36 800 boys [51.1%; 95% CI, 50.2%-52.0%]; 50 219 non-Hispanic white [51.4%; 95% CI, 50.6%-52.3%]) were included in our final analytical sample, of whom 5687 (7.9%; 95% CI, 7.5%-8.4%) had asthma and 11 426 (15.3%; 95% CI, 14.7%-16.0%) had at least 1 disability. Overall asthma prevalence estimates were 10 percentage points higher in children with a disability (16.1%; 95% CI, 14.3%-17.8%) vs children without a disability (6.5%; 95% CI, 6.0%-6.9%). The odds of asthma were significantly higher in children with a disability (odds ratio [OR], 2.77; 95% CI, 2.39-3.21) or delay (OR, 2.22; 95% CI, 1.78-2.77) vs typically growing children. Adjusted models remained significant for all disability categories (overall adjusted OR, 2.21; 95% CI, 1.87-2.62). Subgroup analyses showed ethnic minorities had a higher prevalence of concurrent asthma and developmental disabilities vs non-Hispanic whites (19.8% [95% CI, 16.6%-23.0%] vs 12.6% [95% CI, 11.1%-14.0%]; *P* < .001).

**Conclusions and Relevance:**

These results suggest that US children with various developmental disabilities or delay may have higher odds for developing asthma vs their typically developing peers. These findings support asthma screening in pediatric health care settings among patients with developmental disabilities and delays, particularly among those from ethnic minority backgrounds. In addition, very young children with asthma should be screened for disabilities and delays, because temporality cannot be determined by the current data source and analytical approach.

## Key Points

**Question:**

What are the prevalence estimates of asthma in the US pediatric population aged 0 to 17 years for those with various developmental disabilities and delays?

**Findings:**

In this cross-sectional study of 71 811 participants, overall asthma prevalence estimates were 10 percentage points higher in children with a disability (approximately 16%) vs children without a disability (approximately 6%). The odds of asthma were significantly higher in children with vs without a disability or delay.

**Meaning:**

These findings suggest that asthma screening among patients with disabilities in pediatric health care settings may improve the quality of life and lessen the economic burden among families due to undiagnosed asthma.

## Teaser

This cross-sectional study estimates rates of prevalence of asthma among a diverse sample of US children and adolescents with developmental delay and/or disability.

## Introduction

Asthma is one of the most common chronic diseases in the world among children.^1^ At present, 6 million children (approximately 8%) living in the United States are diagnosed with asthma, incurring $81.9 billion in annual health care expenditures.^2,3^ Moreover, non-Hispanic black male youths in the United States are disproportionally diagnosed with childhood asthma vs their female and non-Hispanic white counterparts.^2^

Concurrently, 53 million (approximately 9%) of the world’s children have a developmental disability.^4^ In 2019, approximately 1 in 6 US children (approximately 17%) from diverse racial and ethnic backgrounds were reported to have a developmental disability.^5^ Developmental disabilities are defined as a group of chronic conditions owing to an impairment in various areas that may include physical, learning, language, and behavioral limitations resulting in functional challenges.^6^ Developmental disabilities are typically categorized as follows: (1) behavioral disorders, including attention-deficit/hyperactivity disorder (ADHD) and autism spectrum disorders (ASD); (2) motor disabilities, including cerebral palsy and seizure; (3) vision, hearing, and speech disabilities; and (4) cognitive disabilities, including intellectual disability (characterized by significant limitations in both intellectual functioning and adaptive behavior, including conceptual, social, and practical skills) and learning disability.^6,7^ Developmental delay is defined as not meeting growth milestones with unknown cause of the delay.^6,7^

The concurrent prevalence of asthma among children and adolescents with various developmental disabilities and delays has received some attention. One systematic review and meta-analysis,^8^ including pediatric and adult samples, reported that individuals with ADHD had a 50% higher odds of having concurrent asthma compared with those without ADHD after adjusting for multiple confounders (pooled adjusted odds ratio [AOR], 1.53; 95% CI, 1.41-1.65; *I*^2^ = 50.76). However, only 5 citations included in this meta-analysis were US population based, and most (4 of 5) only included ADHD as the primary outcome. In addition, in another meta-analysis, Miyazaki and colleagues^9^ reported that ADHD and asthma were associated in children and adolescents in particular. Although Cortese et al^8^ did not compare findings by race and ethnicity, other studies^10,11^ have shown that race/ethnicity is an important effect modifier for asthma risk. Other studies^12,13,14,15^ have reported that children with other disabilities, including ASD, developmental delay, learning disability, and hearing and speech problems, had greater odds of having asthma vs children without disabilities. Conversely, another meta-analysis that included 175 406 pediatric participants^16^ found no significant association between asthma and ASD (pooled OR, 1.26; 95% CI, 0.98-1.61; *I*^2^ = 65.0%).

In summary, previous studies examining the prevalence of asthma among various disability and delay groups have been generally limited to behavioral and cognitive disorders, with mixed results. To help fill this gap in the literature, we report herein the most recent asthma prevalence estimates for 10 developmental disability and delay categories using a population-based survey among the US pediatric population. Owing to the cross-sectional design of the current analysis, results cannot infer causality but are a first step to establishing an association between the 2 health/medical conditions. Based on current reports in the literature, we hypothesized that asthma prevalence estimates would be higher among children and adolescents with various developmental disabilities or delay vs those without disabilities or delay.

## Methods

Our report follows the Strengthening the Reporting of Observational Studies in Epidemiology (STROBE) reporting guideline. The institutional review board and ethics committee at the University of Texas Health Science Center ruled this study to be exempt from review and informed consent because of the use of publicly available, deidentified data for analysis.

### Data Source and Sample Design

A total of 71 811 families with children and adolescents aged 0 to 17 years (hereinafter referred to as children) who participated in the 2016 and 2017 National Survey of Children’s Health (NSCH) were included in this cross-sectional study. The NSCH is a population-based, nationally representative survey directed by the US Health Resources and Services Administration Maternal and Child Health Bureau to assess physical and mental health of US children. The NSCH was conducted every 4 years by using telephone questionnaires from 2003 to 2012. In 2016, the NSCH was substantially revised to merge the current needs and topics of pediatric health and is now conducted through online and mail-based surveys every year. US households were randomly sampled and selected to complete an initial screening survey, and then a main child-specific questionnaire was delivered to eligible households with children. The parents can randomly choose one of their children to complete the second survey. Data were collected from June 10, 2016, to February 10, 2017, for the 2016 survey and from August 10, 2017, to February 10, 2018, for the 2017 survey.

To obtain population-based estimates, each child was assigned a weight, which was composed of a base sampling weight and adjustment of both screener and topical nonresponse, selection of a single child within the respondent household, and various characteristics of children in different states. The overall weighted response rate was 40.7% (n = 50 212) for 2016 and 37.4% (n = 21 599) for 2017.

### Measurements and Assessment

#### Asthma

Childhood asthma diagnosis is reported by parents. Two items from the questionnaire were used to assess child asthma status: “Has a doctor or other health care provider ever told you that this child has asthma?” and, if yes, “Does the child currently have the condition?” We included 62 352 participants (88.2%; 95% CI, 7.5%-8.4%) who never had asthma (answered “no” to both questions) and 3045 (3.9%; 95% CI, 3.6%-4.1%) participants who ever had an asthma diagnosis, but no current condition (answered “yes” and “no,” respectively). If the participant answered “yes” to both questions, he or she was categorized as having current asthma, which was the primary dependent variable of interest and constituted 5687 (7.9%; 95% CI, 7.5%-8.4%) children in this sample. The primary dependent variable was chosen to be consistent with Centers for Disease Control and Prevention reports of current asthma prevalence.^2^

#### Disability Status

Parents were asked if their child had 1 or more developmental disabilities, including (1) behavioral disorders (ADHD or ASD); (2) motor disabilities (cerebral palsy or seizures); (3) vision, hearing, and speech disabilities; (4) cognitive disabilities (intellectual or learning disability); or (5) unspecified developmental delay. Among these, blindness and hearing loss were each assessed by a single item (“Does this child have blindness or problems with seeing, even when wearing glasses?” and “Does this child have deafness or problems with hearing?”), whereas the measures for the other 8 disorders are constructed based on 2 questions: First, “Has a doctor or other health care provider ever told you that this child has the condition?” and second, “If yes, does this child currently have the condition?” The primary independent variable of interest was self-reported disability status (yes or no); a total of 11 426 participants (15.3%; 95% CI, 14.7%-16.0%) answered “yes” in response to this question.

### Covariates

Selected covariates include child age, sex, race/ethnicity, parental educational level, family income, and birth weight. All independent variables were selected based on their significant association with asthma diagnosis and some disabilities reported in previous studies.^8,9,12,13,14,15^

### Statistical Analysis

Data were analyzed from September 20, 2019, to April 5, 2020. Weighted asthma prevalence estimates and 95% CIs were generated for children with and without disabilities, respectively. We used χ^2^ analysis to compare the prevalence rates between these 2 disability groups. Moreover, children were stratified by sex and race to further assess whether asthma prevalence estimates by developmental disabilities differ by sex and white vs nonwhite race/ethnicity. Univariate logistic regression models generated crude ORs to estimate current asthma prevalence for various disabilities. Multivariable logistic regression analysis generated the AORs of an asthma diagnosis after controlling for key demographics (child age, sex, race/ethnicity, parental educational level, and family income) and birth weight. We also performed a sensitivity analysis to explore the association between children who ever had an asthma diagnosis (lifetime asthma) and developmental disabilities compared with those who never had asthma diagnosis. All statistical analyses included the complex sampling plan (strata, cluster, and weight) provided in the NSCH SAS codebook^17^ and were performed in SAS, version 9.4 (SAS Institute, Inc). We used χ^2^ tests and 2-sample *t* tests to compare proportions and means, respectively, and 2-sided *P* < .05 was considered statistically significant.

## Results

A total of 71 811 participants (mean [SE] age, 8.6 [0.1] years; 35 011 girls [48.9%; 95% CI, 48.0%-29.8%] and 36 800 boys [51.1%; 95% CI, 50.2%-52.0%]; 50 219 non-Hispanic white [51.4%; 95% CI, 50.6%-52.3%]) were included in our final analytical sample, of whom 5687 (7.9%; 95% CI, 7.5%-8.4%) had asthma and 11 426 (15.3%; 95% CI, 14.7%-16.0%) had at least 1 disability (Table 1). The 3 most prevalent disabilities were ADHD (8.8%; 95% CI, 8.3%-9.3%), learning disabilities (7.0%; 95% CI, 6.5%-7.5%), and speech problems (5.5%; 95% CI, 5.0%-5.9%) among study respondents representative of US children. Cerebral palsy was the least common disability (0.2%; 95% CI, 0.2%-0.3%). The prevalence of those reporting having at least 2 disabilities in 1 child varied by disability category. For example, 34 children with ADHD (0.6%; 95% CI, 0.3%-0.9%) also had cerebral palsy, whereas 645 children with intellectual disability (93.4%; 95% CI, 90.9%-96.9%) also reported having a learning disability (eTable in the Supplement).

**Table 1.** Participant Characteristics^a^

Characteristic	Unweighted sample size	Weighted % (95% CI)
**Demographic**		
Age, y		
0-5	20 665	32.3 (31.5-33.1)
6-11	21 539	33.9 (33.0-34.7)
12-17	29 617	33.8 (33.1-34.6)
Sex		
Female	35 011	48.9 (48.0-49.8)
Male	36 800	51.1 (50.2-52.0
Race/ethnicity		
White	50 219	51.4 (50.6-52.3)
Nonwhite	21 592	48.6 (47.7-49.4)
Highest educational level in the household^b^		
College degree or higher	44 332	49.1 (48.2-50.0)
Some college or technical school	16 049	22.4 (21.7-23.1)
High school degree or GED	8690	19.6 (18.8-20.4)
Less than high school	1543	8.9 (8.1-9.7)
Family income, % FPL		
≥400	30 834	30.1 (29.4-30.8)
200-399	22 073	27.0 (26.2-27.7)
100-199	11 253	21.7 (20.9-22.5)
0-99	7651	21.2 (20.4-22.1)
Birth weight, g^c^		
<1500	879	1.7 (1.4-1.9)
1500-2499	4549	7.6 (7.1-8.1)
≥2500	62 046	90.7 (90.1-91.3)
Asthma^d^		
Currently have asthma	5687	7.9 (7.5-8.4)
Ever had, no current condition	3045	3.9 (3.6-4.1)
Never had asthma	62 352	88.2 (87.7-88.7)
**Developmental disabilities**		
Behavioral disorders		
ADHD	6115	8.8 (8.3-9.3)
ASD	1679	2.8 (2.4-3.2)
Motor disabilities		
Cerebral palsy	209	0.2 (0.2-0.3)
Seizure	458	0.5 (0.4-0.6)
Vision, hearing, and speech disabilities		
Blindness	894	1.6 (1.3-1.8)
Hearing loss	830	1.3 (1.0-1.5)
Speech problem	3026	5.5 (5.0-5.9)
Cognitive disabilities		
Intellectual disability	699	1.2 (1.0-1.4)
Learning disability	4315	7.0 (6.5-7.5)
Developmental delay	3 149	5.2 (4.9-5.7)
Overall^e^	11 426	15.3 (14.7-16.0)

Abbreviations: ADHD, attention-deficit/hyperactivity disorder; ASD, autism spectrum disorders; FPL, federal poverty level; GED, General Education Diploma.

^a^ Data are from the 2016-2017 National Survey of Children’s Health (n = 71 811).

^b^ Data were missing for 1197 children.

^c^ Data were missing for 4337 children.

^d^ Data were missing for 727 children.

^e^ Includes children with any disability or delay listed above.

Overall, asthma prevalence estimates were significantly higher in children with a disability compared with their peers without disabilities (Table 2). Among children with at least 1 disability, 16.1% (95% CI, 14.3%-17.8%) also reported a concurrent asthma condition, whereas 6.5% of children without disabilities (95% CI, 6.0%-6.9%) had concurrent asthma (*P* < .001). Specifically, the widest asthma prevalence disparity was seen among children with vs those without hearing loss (22.0%, [95% CI, 13.2%-30.7%] vs 7.7% [95% CI, 7.3%-8.2%], respectively; *P* < .001). Similarly, at least 1 in 5 children with cerebral palsy (21.9%; 95% CI, 11.7%-32.0%) or a learning disability (20.0%; 95% CI, 16.6%-23.4%) reported concurrent asthma vs approximately 1 in 12 children without cerebral palsy (7.9%; 95% CI, 7.4%-8.4%) or a learning disability (8.2%; 95% CI, 7.7%-8.7%) (*P* < .001 for both comparisons). Similar trends were also found among children with ADHD, ASD, developmental delay, intellectual disability, seizures, and speech problems. In Table 2, subgroup analysis compared children with and without developmental disabilities by sex and showed that compared with children without developmental disabilities, both boys (7.2% [95% CI, 6.5%-7.9%] vs 16.5% [95% CI, 14.3%-18.8%]) and girls (5.7% [95% CI, 5.2%-6.3%] vs 15.2% [95% CI 12.4%-18.1%]) with any disabilities had significantly higher asthma prevalence estimates (*P* < .001 for both comparisons).

**Table 2.** Weighted Current Asthma Prevalence by Developmental Disability Category^a^

Disability	Weighted % (95% CI)	
	Never had disability	Currently have disability
Behavioral disorders		
ADHD	8.1 (7.5-8.7)	17.2 (15.1-19.3)
Female	7.2 (6.5-7.9)	16.7 (12.9-20.4)
Male	9.0 (8.1-9.9)	17.4 (14.9-19.9)
ASD	8.7 (8.2-9.2)	17.7 (11.7-23.7)
Female	7.7 (7.0-8.4)	14.4 (5.6-23.2)
Male	9.7 (8.9-10.5)	18.5 (11.4-25.7)
Motor disabilities		
Cerebral palsy	7.9 (7.4-8.4)	21.9 (11.7-32.0)
Female	6.8 (6.2-7.4)	18.5 (4.4-32.7)
Male	8.9 (8.2-9.7)	23.6 (10.1,37.2)
Seizure	7.9 (7.4-8.3)	18.3 (11.6-2.9)
Female	6.8 (6.2-7.4)	16.0 (7.3-24.8)
Male	8.9 (8.1-9.6)	20.3 (10.5-30.1)
Vision, hearing, and speech disabilities		
Blindness	7.8 (7.4-8.3)	14.1 (9.6-18.6)
Female	6.8 (7.1-17.5)	12.3 (6.1-7.3)
Male	8.9 (8.1-9.6)	15.9 (8.8-23.0)
Hearing loss	7.7 (7.3-8.2)	22.0 (13.2-30.7)
Female	6.6 (6.1-7.2)	21.3 (7.5-35.0)
Male	8.8 (8.1-9.5)	22.6 (11.9-33.2)
Speech problem	8.5 (8.0-9.1)	16.9 (13.7-20.6)
Female	7.5 (6.8-8.2)	14.8 (5.5-12.7)
Male	9.6 (8.7-10.4)	17.9 (12.9-22.8)
Cognitive disabilities		
Intellectual disability	8.9 (8.3-9.5)	17.5 (11.3-23.7)
Female	7.7 (7.1-8.4)	15.9 (7.5-24.2)
Male	10.0 (9.2-10.9)	18.3 (10.0-26.7)
Learning disability	8.2 (7.7-8.7)	20.0 (16.6-23.4)
Female	7.2 (6.6-7.9)	18.6 (13.8-23.5)
Male	9.2 (8.4-10.0)	20.7 (16.2-25.3)
Developmental delay	8.4 (7.9-9.0)	17.0 (14.0-20.0)
Female	7.6 (6.9-8.2)	14.0 (10.1-17.8)
Male	9.3 (8.5-10.1)	18.5 (14.5-22.4)
Overall^b^		
All patients	6.5 (6.0-6.9)	16.1 (14.3-17.8)
Female	5.7 (5.2-6.3)	15.2 (12.4-18.1)
Male	7.2 (6.5-7.9)	16.5 (14.3-18.8)

Abbreviations: ADHD, attention-deficit/hyperactivity disorder; ASD, autism spectrum disorders.

^a^ Data are representative of the US pediatric population from 2016 to 2017.

^b^ Includes children with any disability or delay listed above.

Overall asthma prevalence estimates were significantly higher in ethnic minority children vs non-Hispanic white children with a developmental disability (19.8% [95% CI, 16.6%-23.0%] vs 12.6% [95% CI, 11.1%-14.0%]; *P* < .001). Specifically, a significantly higher concurrent asthma rate was found in ethnic minority children vs non-Hispanic white children with the following disabilities: ADHD (21.0% [95% CI, 17.0%-25.0%] vs 14.3% [95% CI, 12.2%-16.3%]; *P* = .002), hearing loss (29.4% [95% CI,15.2%-43.7%] vs 11.7% [95% CI, 6.4%-16.9%]; *P* < .001), speech problem (20.6% [95% CI, 14.4%-26.8%] vs 12.8% [95% CI, 9.6%-15.9%]; *P* = .04), and learning disability (24.3% [95% CI, 18.3%-30.3%] vs 15.4% [95% CI, 12.4%-18.3%]; *P* = .004). In contrast, a significantly larger number of non-Hispanic white children had visual impairment and asthma concurrently compared with their ethnic counterparts (20.7% [95% CI, 12.5%-29.0%] vs 9.6% [95% CI, 5.6%-13.6%]; *P* = .02) (eFigure in the Supplement).

Crude logistic regression analysis showed a significant association between asthma and all disability categories in US children (Figure, A). In general, children who had at least 1 disability had almost 3 times higher odds of having reported current asthma conditions compared with typically developing children (OR, 2.77; 95% CI, 2.39-3.21; *P* < .001). Notably, the odds of concurrent asthma increased the most (more than 3-fold) among children with cerebral palsy (OR, 3.27; 95% CI, 1.80-5.94; *P* < .001) or hearing loss (OR, 3.35; 95% CI, 2.01-5.60; *P* < .001). Subsequently, the odds of asthma was twice as high or nearly twice as high in children with vs without the following disabilities: (1) behavioral disabilities consisting of ADHD (OR, 2.36; 95% CI, 1.99-2.78) and ASD (OR, 2.25; 95% CI, 1.48-3.41); (2) motor disability consisting of seizure (OR, 2.63; 95% CI, 1.68-4.13); (3) blindness (OR, 1.93; 95% CI, 1.32-2.81) and speech problem (OR, 2.19; 95% CI, 1.67-2.87); and (4) cognitive disabilities consisting of intellectual disability (OR, 2.17; 95% CI, 1.41-3.35) and learning disability (OR, 2.80; 95% CI, 2.24-3.49). Children with developmental delays were also at more than a 2-fold higher odds of concurrent asthma (OR, 2.22; 95% CI, 1.78-2.77).

**Figure.**
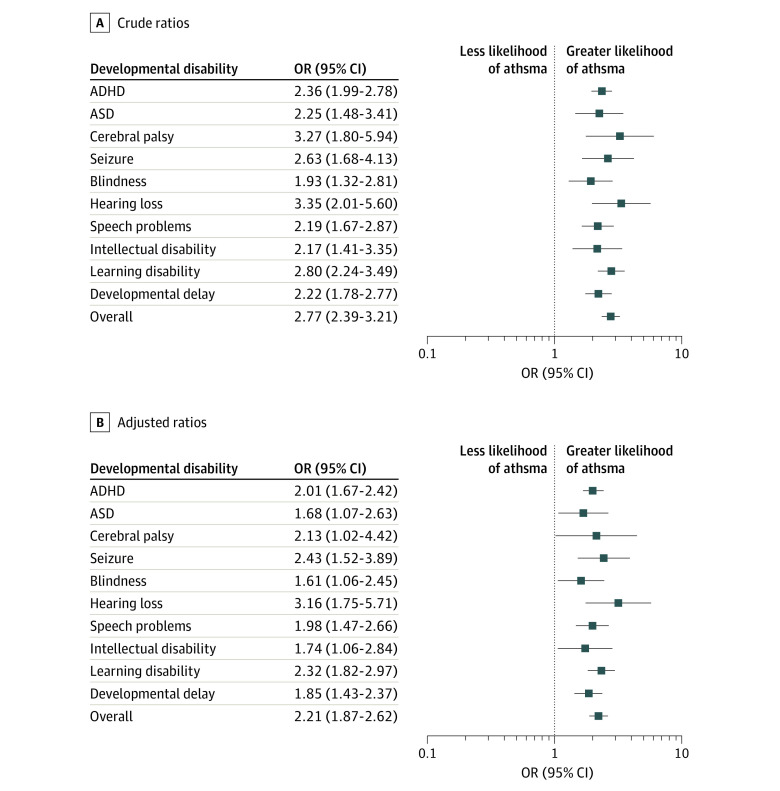
Odds of Current Asthma Among the US Pediatric Population With Developmental Disabilities, 2016-2017 Data are shown as crude odds ratios (ORs) (A) and multivariable logistic regression adjusted for age, sex, race/ethnicity, educational level, family income, and birth weight (B). ADHD indicates attention-deficit/hyperactivity disorder; ASD, autism spectrum disorders. Overall category includes children with any of the 10 disabilities or delay. Error bars indicate 95% CIs.

All associations remained significant for all disability categories after adjusting for demographic characteristics and birth weight (Figure, B). Overall, children with disabilities had 2 times higher odds of having concurrent asthma vs their peers with no disabilities (AOR, 2.21; 95% CI, 1.87-2.62). Even after adjustment, children with hearing loss had the highest odds of concurrent asthma (AOR, 3.16; 95% CI, 1.75-5.71). A sensitivity analysis using the multivariable logistic regression showed the association remained significant between lifetime asthma and a co-occurring disability (AOR, 1.91; 95% CI, 1.65-2.20).

## Discussion

We reported herein the prevalence of asthma among children with at least 1 of the 10 developmental disabilities or delay reported in the most recent NSCH population health data set. Results showed that approximately 1 in 6 children (approximately 16%) with a disability or developmental delay had concurrent asthma vs about 1 in 16 (approximately 6%) children without any disability or delay. These results remained even after model adjustment for key demographic variables and birthweight. Analyses illustrated the variability in concurrent asthma prevalence by various disability categories as well, ranging from 2 to 3 times the odds compared with children with no disability. Moreover, ethnic minorities had higher odds of concurrent asthma with a disability or delay vs their non-Hispanic white counterparts.

Few previous studies have used population-based data to examine the associations between asthma and developmental disabilities and delays. Although our results showed a consistent and strong association between disability, regardless of category, and asthma, no clear categorical patterns emerged. For example, in the group with vision, hearing, or speech disabilities, children with hearing loss had the highest odds of concurrent asthma, whereas blind children had the lowest odds, posing challenges to future studies dedicated to determining potential causal pathways. Other studies have postulated that among those with ADHD specifically, inflammation may be a link with asthma.^18,19^ Specifically, patients with ADHD showed increased stress level, leading to a neuroimmunological response that eventually triggers the co-occurrence of asthma.^18,19^ Furthermore, shared risk factors between ADHD and asthma, such as genetic or prenatal stress, could be another possible explanation for the increased susceptibility of concurrent conditions.^20,21^ Also, inhaled corticosteroids, a common pharmacological treatment for asthma, may cause neurological adverse effects that can be misclassified as ADHD in some patients. However, a large population-based study did not find that asthma medications increased the odds of ADHD.^22^

The association between childhood asthma and cognitive disabilities may be intermediated by asthma-related missed school days, especially among minority children. Nearly half of all children with asthma report missing days of school in the United States.^23^ School absenteeism has been linked with low academic performance, which in turn may be misclassified as a co-occurring learning disability.^23^ Indeed, a particular challenge to educating children with various cognitive disabilities and concurrent asthma is adequate disease management, such as appropriate use of inhalers, anticipation of an asthmatic episode, and anxiety due to this anticipation.

Very few previous studies in the literature documented concurrent asthma prevalence among children with motor disabilities, such as cerebral palsy or seizure, although children with neurological impairment have been reported to often present with chronic respiratory problems.^24,25^ For example, 58% of patients with cerebral palsy have also been reported to have a daily cough or wheeze in Australia.^26^ Anoxic tonic seizures were reported as a complication of severe asthma in one case series,^27^ whereas another small retrospective study concluded that there was no association between childhood asthma and seizure.^28^ The association between asthma and motor disabilities could be explained by the acute nonischemic hypoxia associated with asthma. When having an asthma attack, patients tend to inhale more nitrogen and less oxygen, which in turn induces activation of both focal and generalized epilepsy.^27^

Notably, results herein showed that children in the hearing-loss group were at the highest odds for concurrent asthma. However, this group also had a high variability as seen from the error bars in the Figure. This finding is supported by a report of a randomized clinical trial^29^ to examine auditory functions among adults with chronic asthma. The authors reported that hearing loss, especially in high frequencies, presented more often than expected among individuals with concurrent asthma.^29^ The etiology of hearing loss could also include systemic hypoxia associated with asthma. Specifically, hypoxia decreases cerebral blood flow and causes inadequate blood supply in the cochlea and eventually leads to the development of hearing loss.^30,31^

Another key finding of our study was that US ethnic minority children are more likely to have concurrent asthma and developmental disabilities or delays compared with non-Hispanic white children. The prevalence, morbidity, and mortality of asthma is well documented to be higher in ethnic minority children.^32,33^ This prevalence is likely owing to multisystem dynamic effects, such as the differences in the quality of support and education available to the 2 groups. Health care, health insurance, and housing are additional potential explanatory factors for this finding. For example, non-Hispanic black children are 2 times more likely to have an asthma diagnosis, 4 times more likely to have asthma-related hospitalization, and 8 times more likely to die prematurely because of an asthma attack.^32,33^ Similarly, in the United States, non-Hispanic black children are more likely to be diagnosed with a developmental disability compared with non-Hispanic white children.^5^ However, the variance in concurrent diagnoses of asthma and developmental disabilities among ethnic groups has not been previously described, even in smaller clinical samples.

Findings herein could promote the discussion of challenges to quality of life among children with asthma and disabilities, because the burden of health care needs increases among their clinicians and caregivers.^34^ Indeed, Koehler et al^34^ have reported depressive symptoms are more common in caregivers of children with both asthma and disabilities compared with caregivers of typically growing children without asthma. Even more striking, current pediatric asthma guidelines do not list disability or delay as a risk factor (for asthma).^35,36^ Furthermore, uncontrolled asthma symptoms in the United States may lead to nearly $1 billion in direct and indirect health care expenditure in the next 20 years.^37^ Hence, raising awareness about the increased odds of a concurrent diagnosis among both patient groups (disability and asthma) could have significant clinical implications, leading to reduced diagnostic delay, improved respiratory health management among children with disabilities, and reduced health care costs.

### Limitations

This study had some limitations. As mentioned above, we could not establish a temporal or a causal relationship because of the cross-sectional nature of our study. Parent report of disability and/or asthma may be prone to report or recall bias, but our prevalence estimates were similar to those of other published reports,^1,4,5^ indicating the accuracy and consistency of our data source. Although our analysis comprehensively controlled for key parental sociodemographic factors and child birth weight, other potential confounders that should be considered may exist, such as maternal age at birth^8^ and missed school days^23^; however, we were unable to include these owing to data unavailability. Multiple function domains are often affected among children with disabilities because of the nature and extent of brain impairment or increased susceptibility to other causes of disability (eg, malnutrition, trauma, infection).^6^ Therefore, the likelihood of having multiple disabilities is high, which may lead to overestimation of an association between asthma and a single disability. Also, medical surveillance bias may exist in that children with 1 condition may be more likely to be diagnosed with a concurrent condition. Finally, the moderate response rates (40.7% for 2016 and 37.4% for 2017) may lead to selection bias; however, the well-calibrated sample weights assigned by the NSCH have been created to decrease possible sample bias.

## Conclusions

This study is, to our knowledge, one of the most comprehensive analyses examining the association between various disability categories and asthma in US population-level data. Results showed that asthma prevalence estimates were 10 percentage points higher in children with vs without a disability (16% vs 6%, respectively). Children from a minority background in particular had higher odds of having concurrent asthma and developmental disabilities. The odds of asthma were nearly 3-fold in children with a disability and 2-fold among children with a developmental delay vs their peers with no disabilities or delay. These results can inform pediatric clinical practice about the importance of screening for asthma among patients with various developmental disabilities or delay, which may increase the quality of life and decrease the economic burden due to undiagnosed asthma or disabilities. A simple screener asking parents about breathing patterns, coughing, wheezing, and other risk factors may help identify an asthma diagnosis earlier so that appropriate medical and/or medication approaches can be addressed with the family. In the future, longitudinal studies rigorously controlling for possible maternal and child confounders are needed to explore the possible link between asthma and developmental disabilities in a diverse pediatric population.

## Notes

Supplement. **eTable.** Weighted Percentage Estimates for Children With Concurrent Disabilities, NSCH 2016-2017 (n = 71 811) **eFigure.** Weighted Prevalence of Asthma by Disability Category and Race in US Children, 2016-2017
