# Supplementary material for: Estimated Prevalence of Asthma in US Children With Developmental Disabilities
Source: JAMA Netw Open. 2020 Jun 16;3(6):e207728. doi: 10.1001/jamanetworkopen.2020.7728 (PMC7298611; doi:10.1001/jamanetworkopen.2020.7728)
Supplement: Supplement. — eTable. Weighted Percentage Estimates for Children With Concurrent Disabilities, NSCH 2016-2017 (n = 71 811) eFigure. Weighted Prevalence of Asthma by Disability Category and Race in US Children, 2016-2017 [file jamanetwopen-3-e207728-s001.pdf]

Supplementary Online Content

Xie L, Gelfand A, Delclos GL, Atem FD, Kohl HW III, Messiah SE. Estimated prevalence of asthma in US children with developmental disabilities. *JAMA Netw Open*. 2020;3(6):e207728. doi:10.1001/jamanetworkopen.2020.7728

eTable. Weighted Percentage Estimates for Children With Concurrent Disabilities, NSCH 2016-2017 (n = 71 811)

eFigure. Weighted Prevalence of Asthma by Disability Category and Race in US Children, 2016-2017

This supplementary material has been provided by the authors to give readers additional information about their work.

©2020 Xie L et al. *JAMA Network Open*.

eTable. Weighted Percentage Estimates for Children With Concurrent Disabilities, NSCH 2016-2017 (n = 71 811)

	ADHD	ASD	CP	Seizure	Blindness	Hearing loss	Speech problem	ID	LD	DD
**ADHD**	6115 (100%)	763 (14.2%)	34 (0.6%)	107 (1.9%)	164 (5.1%)	150 (2.6%)	775 (14.3%)	267 (4.9%)	2163 (37.7%)	1267 (22.0%)
**ASD**	763 (14.2%)	1679 (100%)	22 (1.6%)	88 (5.1%)	65 (3.2%)	60 (2.2%)	742 (48.1%)	300 (19.7%)	922 (61.5%)	1041 (62.3%)
**CP**	34 (0.6%)	22 (1.6%)	209 (100%)	69 (28.1%)	69 (31.8%)	29 (17.1%)	117 (52.7%)	88 (51.7%)	127 (62.7%)	152 (75.4%)
**Seizure**	107 (1.9%)	88 (5.1%)	69 (28.1%)	458 (100%)	76 (15.4%)	32 (6.9%)	163 (40.2%)	120 (27.1%)	208 (49.3%)	216 (50.7%)
**Blindness**	164 (5.1%)	65 (3.2%)	69 (31.8%)	76 (15.4%)	894 (100%)	105 (10.7%)	196 (17.4%)	110 (10.5%)	261 (28.6%)	247 (23.4%)
**Hearing loss**	150 (2.6%)	60 (2.2%)	29 (17.1%)	32 (6.9%)	105 (10.7%)	830 (100%)	213 (27.4%)	86 (7.2%)	204 (27.2%)	201 (27.8%)
**Speech problem**	775 (14.3%)	742 (48.1%)	117 (52.7%)	163 (40.2%)	196 (17.4%)	213 (27.4%)	3026 (100%)	521 (14.4%)	1430 (46.4%)	1669 (52.4%)
**ID**	267 (4.9%)	300 (19.7%)	88 (51.7%)	120 (27.1%)	110 (10.5%)	86 (7.2%)	521 (14.4%)	699 (100%)	645 (93.4%)	656 (90.1%)
**LD**	2163 (37.7%)	922 (61.5%)	127 (62.7%)	208 (49.3%)	261 (28.6%)	204 (27.2%)	1430 (46.4%)	645 (93.4%)	4315 (100%)	2147 (50.7%)
**DD**	1267 (22.0%)	1041 (62.3%)	152 (75.4%)	216 (50.7%)	247 (23.4%)	201 (27.8%)	1669 (52.4%)	656 (90.1%)	2147 (50.7%)	3149 (100%)

NSCH, National Survey of Children's Health

1) behavioral disorders: ADHD, Attention-deficit/hyperactivity disorder; ASD, Autism Spectrum Disorders; 2) motor disabilities: CP, Cerebral Palsy; seizure; 3) vision, hearing and speech disabilities; 4) cognitive disabilities: ID, intellectual disability; LD, learning disability; 5) DD, developmental delay

©2020 Xie L et al. *JAMA Network Open*.

eFigure. Weighted Prevalence of Asthma by Disability Category and Race in US Children, 2016-2017

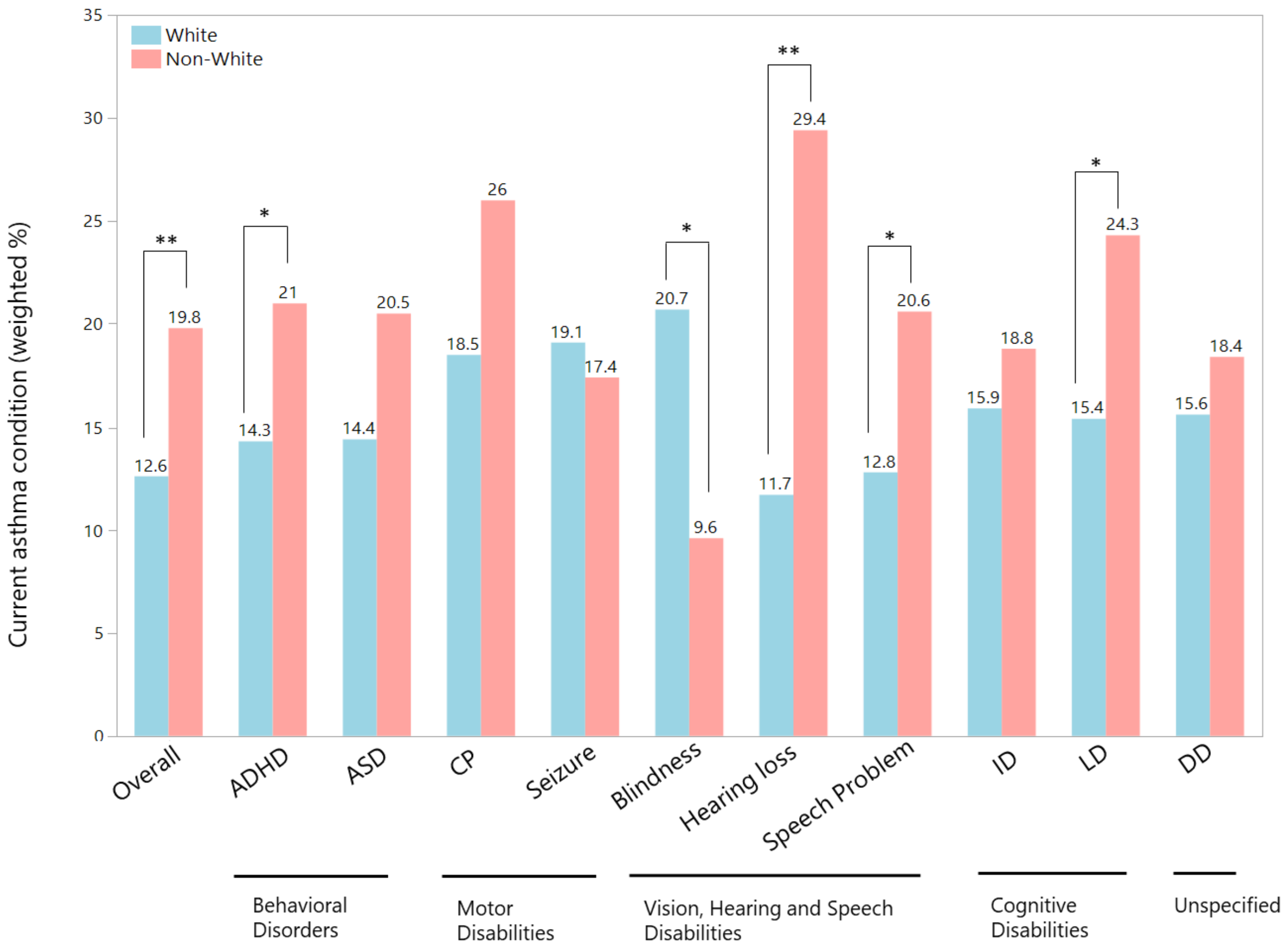

ADHD, Attention-deficit/hyperactivity disorder; ASD, Autism Spectrum Disorders; CP, Cerebral Palsy; ID, intellectual disability; LD, learning disability; DD, developmental delay; Overall, children with any disabilities, including ADHD, ASD, Cerebral Palsy, seizure, vision, hearing and speech, intellectual, learning disability and developmental delay.

* $P < 0.05$

** $P < 0.001$

©2020 Xie L et al. *JAMA Network Open*.
